# Effects of Four Kinds of Oxide Nanoparticles on Proteins in Extracellular Polymeric Substances of Sludge

**DOI:** 10.1155/2020/1754134

**Published:** 2020-03-02

**Authors:** Weiyun Wang, Ying Zang, Cijia Wang, Kangni Wang, Rundong Li

**Affiliations:** ^1^College of Energy and Environment, Shenyang Aerospace University, Shenyang 110136, China; ^2^Key Laboratory of Clean Energy, Liaoning Province, Shenyang 110136, China

## Abstract

Proteins are the most important component in sludge extracellular polymeric substances (EPS) and play a crucial role in the formation of sludge flocs, adsorption performance of sludge, and flocculation ability of sludge. This research is aimed at exploring the changes in proteins in EPS extracted from concentrated sludge after various nanoparticle (NP) treatments. The results showed that the protein content in EPS decreased by 40% after nanoalumina (Al_2_O_3_ NPs) treatment but increased at varying degrees after nanoferric oxide (Fe_3_O_4_ NPs), nanozinc oxide (ZnO NPs), and nanotitanium dioxide (TiO_2_ NPs) treatments. The four kinds of nanoparticles not only affected the protein content in EPS but also influenced the types and structures of proteins. The results of three-dimensional fluorescence spectroscopy showed that the tyrosine-like protein content in soluble EPS (SEPS) decreased after treatments with four kinds of NPs. Infrared spectroscopy analysis revealed that the absorption intensity of amide I and amide II weakened after Al_2_O_3_ NP treatment, whereas that of amide I enhanced after Fe_3_O_4_ NP, ZnO NP, and TiO_2_ NP treatments. Further analysis of the secondary structure of proteins in the infrared range of 1700–1600 cm^−1^ demonstrated that the value of *α*-helix/(*β*-sheet+random coil) decreased from 0.513 to 0.383 in SEPS after TiO_2_ NP treatment. For the samples treated by Fe_3_O_4_ NPs, the percentage of *α*-helix significantly increased and that of *β*-sheet slightly decreased in proteins from SEPS and loosely bound EPS.

## 1. Introduction

A large number of nanomaterials are released into the environment owing to their wide applications. Kiser et al. firstly reported the emergence and migration characteristics of titanium dioxide nanoparticles (TiO_2_ NPs) in sewage treatment plants and showed that NPs with similar properties would remain in the sludge through adsorption of biomass material [1]. Therefore, new fields of research on the interaction between NPs and extracellular polymeric substances (EPS) in biological wastewater treatment have been developed. EPS is a mixture of polymer accounting for 60%–80% of organic matter in sludge [2] and is usually including proteins, humic acid substances, polysaccharides, nucleic acids, and phospholipids, which are distributed in soluble EPS (SEPS), loosely bound EPS (LB-EPS), and tightly bound EPS (TB-EPS), respectively [3]. The characteristics of EPS affect the structure, sedimentation, flocculation and dewatering performance, and charge of sewage sludge [4].

Proteins (PN) are one of the main components of sludge EPS and play an important role in bacterial cell aggregation, adsorption, and biofilm formation. Some researchers believed that proteins, not polysaccharides and humic substances, played a key role in sludge dewatering performance [5]. However, there are hundreds of proteins with different isoelectric point, molecular weight, and structure in activated sludge [6]. Higgins and Novak [7] demonstrated that the dewatering performance of sludge is mainly affected by the ratio of protein to polysaccharide. When compared with polysaccharides, proteins have more significant effect on the sludge dewatering performance, and high protein/polysaccharide ratio has certain adverse effects on sludge dewatering process. Zhao et al.'s research showed that the presence of diclofenac promoted the EPS content of the EBPR system and increased the ratio of protein to polysaccharide [8]. Some studies have shown that certain metal oxides, such as CuO, CeO_2_, and ZnO, have various effects on the composition of EPS in activated sludge [9, 10]. Some previous study [11, 12] showed that protein concentration increased in SEPS and LB-EPS with the increasing TiO_2_ NP concentration. As long as these NPs exist in the biosphere, they may interact with humans and other organisms in an imperceptible way [13]. Exposure to nanoparticles has become an inevitable phenomenon given their widespread use, and consequently, nanotoxicological evaluations have recently gained a great deal of attention [14]. Under certain conditions, some NPs may destroy cell membranes and cause the release of intracellular substances [15]; however, the presence of EPS may weaken this effect. In addition to its influences on the protein content in EPS, NPs also have certain effects on the types and secondary structures of proteins in EPS. It has been reported that the existence of secondary structures of some proteins is beneficial to cell aggregation, adsorption, and biofilm formation, such as aggregated strands, *β*-sheets, and *α*- and 3-turn helices. On the contrary, some protein structures are not conducive to cell aggregation, adsorption, and biofilm formation, such as antiparallel *β*-structures and random coils [16, 17]. The interaction among extracellular proteins, NPs, and cells is a complex problem. In addition to altering the secondary structure of proteins, NPs can also damage the structure of cells. Accordingly, it is necessary to explore the interaction between EPS and NPs.

In the present study, four types of NPs commonly used in industry are selected, namely, zinc oxide (ZnO NPs), TiO_2_ NPs, alumina oxide (Al_2_O_3_ NPs), and iron oxide (Fe_3_O_4_ NPs), and the quantitative and qualitative changes of proteins in EPS after NP treatment were analyzed. Because these four kinds of NPs are used widely and easily transfer from sewage to sludge, it is more practical to focus on the impact of these four kinds of NPs on the protein in EPS extracted from sludge. The qualitative and quantitative analyses of the effects of four kinds of NPs on protein distribution in EPS were conducted by the analysis of changes of the main functional groups and major fluorescent components in EPS.

## 2. Materials and Methods

### 2.1. Characterization of NPs

The particle size of NPs was 30 nm. The nanopowder used was purchased from Shanghai Dibai Biotechnology Co., Ltd., China. The Al_2_O_3_ NPs used in this experiment are *γ*-Al_2_O_3_.

Scanning Electron Microscopy (SEM) images of the oxide nanoparticles were obtained using a FEI Nova Nano SEM 450 Scanning Electron Microscope with an energy dispersive spectrometer (EDS). The SEM image and EDS spectrum of the oxide nanoparticles were presented in Fig. [Supplementary-material supplementary-material-1]. The X-ray diffraction patterns of oxide nanoparticles were measured at room temperature using the Shimadzu XRD-7000s with Cu K*α* (5°–90°). The XRD pattern of the oxide nanoparticles is shown in Fig. [Supplementary-material supplementary-material-1].

### 2.2. Characterization of Sludge Samples

Sludge samples were collected from Shenyang North Sewage Treatment Plant, China, with a capacity of 400,000 m^3^/d. All the samples were stored in a refrigerator (4°C ± 1°C). The characteristics of the sludge are shown in Table 1.

### 2.3. Treatment of Sludge with NPs

Four typical NPs were selected and applied to mechanically dewatered sludge and concentrated sludge, respectively. The moisture content of dewatered sludge and concentrated sludge was adjusted to 90% and 98%, respectively. Then, the sludge was quickly mixed. To determine the optimal NP dosage, the NPs were added to the samples at different doses (g/g total suspended solid (TSS)). Owing to their aggregation, the NPs were suspended in storage solutions with different concentration gradients. Before each experiment, the NPs were sonicated for 10 min at 18 W. The dosage was regulated by controlling the volume of the storage solution. In a 100 mL centrifuge tube, 1 g (dry basis) of the sludge sample was, respectively, mixed with different doses of NPs and incubated in a shaking incubator at 25°C and 150 rpm for 4 h. All the experiments were performed in triplicate.

### 2.4. EPS Extraction

**Figure 1:**
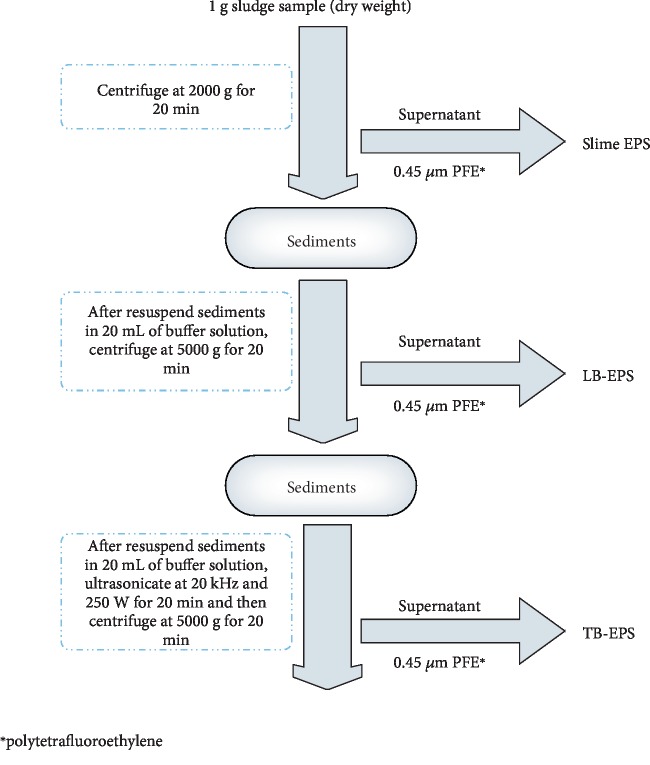
Extraction of different EPS fractions.

In this study, EPS was extracted from the sludge by centrifugation and ultrasonication to further analyze protein distribution. First, 1 g of sludge (dry basis) was added to 100 mL centrifugal tube and centrifuged for 15 min at 2000 × *g*. Then, the supernatant, or SEPS, was collected and filtered through 0.45 *μ*m filter membrane, while the sediment was diluted to 20 mL with buffer and centrifuged for 15 min at 5000 × *g* to obtain the components of LB-EPS in the supernatant. Finally, the residual sediment in the centrifugal tube was suspended in 20 mL of buffer solution, and the TB-EPS was collected by ultrasonication at 250 W for 20 min and centrifugation at 20,000 × *g* for 20 min. The buffer solution used in the experiment contained Na_3_PO_4_, NaH_2_PO_4_, NaCl, and KCl at a molar ratio of 2 : 4 : 9 : 1 (pH = 6.89). The EPS extraction process is shown in Figure 1 [18].

### 2.5. Other Analytical Methods for Investigation of EPS Components

#### 2.5.1. 3D-EEM Analysis

The 3D-EEM spectra were obtained using a Hitachi F-4500 fluorescence spectrophotometer (Hitachi, Japan). The excitation spectrum ranged from 200 to 450 nm, and the emission spectrum ranged from 200 to 550 nm. The emission spectrum was mapped once the excitation wavelength increased by 10 nm. The slit width of both excitation and emission wavelengths was 10 nm, and the scanning speed was 1200 nm/min.

#### 2.5.2. FT-IR Spectroscopic Analysis

The EPS was lyophilized in a vacuum freeze dryer, and 1 mg of the lyophilized powder was mixed with 100 mg of potassium bromide (FT-IR grade), ground thoroughly, and pressed to form a tablet by using a pressure machine. The prepared samples were analyzed by a FT-IR spectrometer (Nicolet iS50, Thermo Fisher, USA). Owing to the abundant information on the secondary structure of proteins in the amide I region, the spectrum band in the amide I region could be easily affected by external conditions. Hence, the amide I band (1700–1600 cm^−1^), which is attributable to the C-O bond stretching vibrations of peptide groups in proteins, was selected to explore the changes in the secondary structure of proteins [19]. The PEAKFIT v4.12 software was used to separate overlapping peaks. The second derivative spectra of the original amide I were obtained using a nine-point Savitzky-Golay derivative function [16, 20]. Subsequently, the amide I band was deconvoluted on the basis of maximum absorption strength, band frequency, and bandwidth of the second derivative spectra [9, 19].

#### 2.5.3. Colorimetric Analysis

The standard curve was constructed based on BSA, protein content in EPS was determined by the Lowry method [21], and DNA content in EPS was ascertained by diphenylamine colorimetry [22]. Calf thymus DNA was used as the standard curve.

## 3. Results and Discussion

### 3.1. Colorimetric Analysis

**Figure 2:**
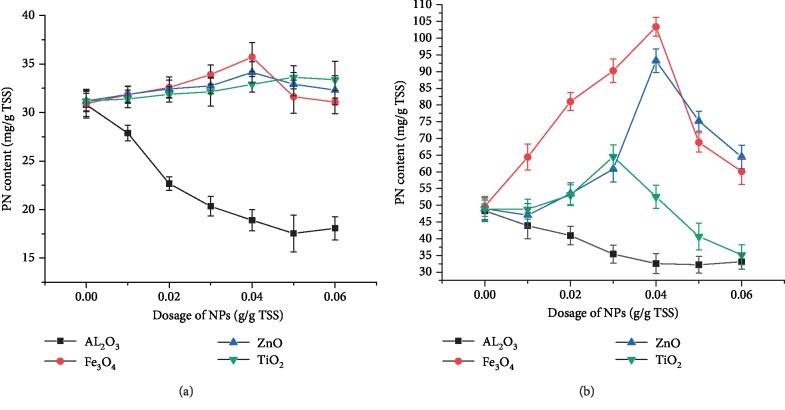
Effect of NPs on the protein content in EPS of mechanical dewatered sludge (a) and concentrated sludge (b).

The effect of NPs on the EPS protein content in the sludge was analyzed.  Figure 2(a) shows the effect of four kinds of NPs (Al_2_O_3_ NPs, Fe_3_O_4_ NPs, TiO_2_ NPs, and ZnO NPs) on the protein content in EPS extracted from mechanically dewatered sludge. It can be indicated in Figure 2 that the PN content decreased with the increase in Al_2_O_3_ NPs, while the protein content increased under the action of Fe_3_O_4_ NPs, TiO_2_ NPs, and ZnO NPs. When compared with the blank group, the PN contents increased by 15%, 10%, and 8% when 0.04 g/g TSS Fe_3_O_4_ NPs, 0.04 g/g TSS ZnO NPs, and 0.05 g/g TSS TiO_2_ NPs were added, respectively; however, the PN contents decreased by 44% when 0.05 g/g TSS Al_2_O_3_ NPs was added. In general, the protein content remained almost constant with the addition of Fe_3_O_4_ NPs, TiO_2_ NPs, and ZnO NPs, whereas the PN contents significantly reduced when Al_2_O_3_ NPs were added.

The effects of four kinds of NPs on the protein content in EPS extracted from concentrated sludge are shown in Figure 2(b). With the increasing dosage of Fe_3_O_4_ NPs, TiO_2_ NPs, and ZnO NPs, the PN content increased; however, the PN content decreased with the increasing Al_2_O_3_ NPs dosage. In particular, the PN content decreased by 40% when 0.04 g/g TSS Al_2_O_3_ NPs were added. These results were similar to the effects of NPs on the PN content in EPS extracted from mechanically dewatered sludge, indicating that the physical interactions between sludge and Al_2_O_3_ NPs may be the main factor affecting the interaction and that physical, rather than chemical, interactions occurred between them. The aggregation of Al_2_O_3_ NPs on the surface of the cells in the sludge reduced the secretion of extracellular polymers.

When 0.01–0.04 g/g TSS Fe_3_O_4_ NPs was added to the sludge, the PN content increased, whereas addition of 0.04–0.06 g/g TSS Fe_3_O_4_ NPs decreased the content of PN. The PN content increased by 110%, reaching the maximum value, when 0.04 g/g TSS Fe_3_O_4_ NPs was added. In a previous study, the presence of rod-shaped bacteria was found to slow down the rapid oxidation of Fe^2+^ to Fe^3+^ and lead to the emergence of acicular iron oxides [23]. As large numbers of total coliforms and fecal coliforms are found in sludge, Fe_3_O_4_ NPs may form acicular products during the oxidation process. These acicular products formed by oxidation may come into physical contact with the bacterial cells, causing puncture damage and resulting in the dissolution of some intracellular proteins.

Addition of 0.01–0.04 g/g TSS ZnO NPs increased the PN content, whereas addition of 0.05–0.06 g/g TSS ZnO NPs decreased the PN content. The PN content increased by 94%, reaching a maximum value, when 0.04 g/g TSS ZnO NPs were added. When 0.01–0.03 g/g TSS TiO_2_ NPs were added, the PN content increased, whereas addition of 0.04–0.06 g/g TSS TiO_2_ NPs decreased the PN content. The PN content reached the maximum, presenting 33% increase, when 0.03 g/g TSS TiO_2_ NPs were added. Brayner et al. [24] found that ZnO NPs can attach to the surface of *Escherichia coli* cells, interact with them, and destroy the cell wall, thus changing the bacterial morphology and releasing the bacterial cell content. The leakage of the cell contents caused by ZnO NPs is presumed to be owing to the mechanical damage of bacteria caused by the rough surface of ZnO NPs [25]. In the present study, the pH of concentrated sludge was 6.8, the isoelectric point for raw powdered ZnO NPs was found to be around pH 9.5 [26], and hence, ZnO NPs are positively charged in the sludge medium; ZnO NPs attract each other to the negatively charged sludge flocs, which cause the zinc oxide nanoparticles to contact with the sludge flocs more frequently, thus making the sludge cells more susceptible to the toxicity of ZnO NPs and the leakage of the cell contents. Therefore, both the electrostatic interactions of physical properties and the toxic effect of chemical properties can promote the dissolution of sludge cell contents and increase the extracellular polymer content.

Maness et al. [27] believed that activated oxygen species produced on the surface of TiO_2_ NPs, such as hydroxyl radical, superoxide anion, and hydrogen peroxide, could degrade the microbial cell membrane, facilitating entry of TiO_2_ NPs into the cell and subsequent photocatalytic oxidation of intracellular substances. However, the decrease in the protein content in EPS resulting from high concentration of NPs could possibly be owing to excessive NPs that tend to agglomerate. In the present study, the pH of concentrated sludge was 6.8; the isoelectric point for TiO_2_ NPs was found to be around pH 6.0 [28]. Therefore, TiO_2_ NPs are negatively charged in the sludge medium. Although NPs and negatively charged sludge repel each other, reducing the collision frequency between sludge cells and NPs, the toxic effect of NPs still causes partial sludge cell contents to dissolve, resulting in a small increase in extracellular protein content.

In a previous study on the affinity of Al_2_O_3_ NPs towards biosolids used in municipal wastewater treatment plants, electrostatic interactions were found to be crucial in the relationship between NPs and organisms [29]. Some studies have shown that TiO_2_ NPs, CeO_2_ NPs, and other NPs attached to the surface of microbial cells can cause damage to cell membranes and inhibit cell activity [30]. It has been reported that the toxicity of Al_2_O_3_ NPs is weaker than that of other types of NPs [30, 31] and that Al_2_O_3_ NPs exhibit only slight toxicity to certain bacteria [32].

**Figure 3:**
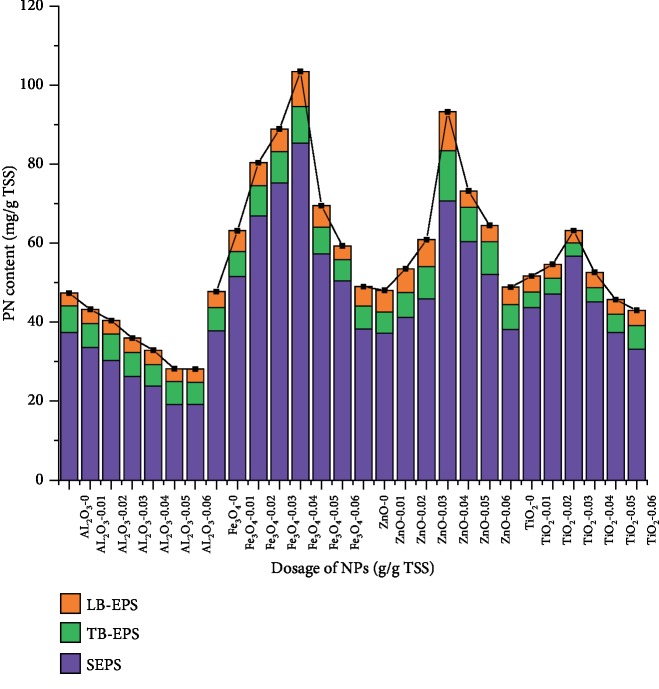
Effect of NPs on protein distribution.

The effect of Al_2_O_3_ NPs on protein distribution in EPS extracted from concentrated sludge is shown in Figure 3. In the present study, the pH of concentrated sludge was 6.8; from the reference, isoelectric point for Al_2_O_3_ NPs was measured to be 8.9 [33]; and hence, Al_2_O_3_ NPs are positively charged in the sludge medium. At a NP dosage range of 0.01–0.04 g/g TSS, the protein content gradually decreased with the increasing dosage of NPs. In particular, the proportion of proteins in SB-EPS gradually decreased, which may be owing to the adherence of positively charged Al_2_O_3_ NPs onto the surface of negatively charged cells, which subsequently resulted in the adsorption of a large number of extracellular proteins, leading to the increase in proteins in LB-EPS and TB-EPS. The decrease in proteins may be related to the release of Al^3+^ from Al_2_O_3_ NPs, causing compression of electric double layer, adsorption bridging, and capture. Although Al_2_O_3_ NPs are slightly soluble in water, they can only be released in acidic medium. In neutral and alkaline media, Al(OH)_3_ precipitates in the NP solution, which reduces Al_2_O_3_ NP concentration in the solution and its toxicity to cells [34]. In conclusion, considering both electrostatic interactions of physical properties and toxicity of chemical properties, electrostatic interactions and adsorption properties of Al_2_O_3_ NPs play a major role in the reaction between sludge and Al_2_O_3_ NPs, while the toxic effects of nanoparticles are ignored.

The effect of Fe_3_O_4_ NPs on protein distribution in EPS extracted from concentrated sludge is shown in Figure 3. With the increasing dosage of Fe_3_O_4_ NPs, the PN content in SEPS, LB-EPS, and TB-EPS increased. In particular, the proportion of proteins in SEPS gradually increased from 0.79 to 0.85, indicating that proteins in TB-EPS and LB-EPS were gradually released into the SEPS. Furthermore, the DNA content in the EPS was determined to explore the destruction of cell membrane. The results revealed that 0.36 mg/g TSS DNA content was detected following the addition of 0.04 g/g TSS Fe_3_O_4_ NPs, whereas the blank sample presented 0.2175 mg/g TSS DNA content. In the present study, the pH of concentrated sludge was 6.8, the isoelectric point for Fe_3_O_4_ NPs was found to be around pH 6.5 [35], and hence, the electrostatic interactions can be ignored. In conclusion, mechanical damage and toxicity of Fe_3_O_4_ NPs to sludge cells play a major role in the reaction of sludge and Fe_3_O_4_ NPs.

Figure 3 illustrates the effect of TiO_2_ NPs and ZnO NPs on protein distribution in the EPS extracted from concentrated sludge. Following the addition of 0.01–0.03 g/g TSS TiO_2_ NPs, the PN content decreased in TB-EPS and LB-EPS but increased in SEPS, suggesting dissolution of proteins from the inner layer to the outermost layer. With the increasing dosage of NPs, the protein distribution was almost the same as that of the blank sample, whereas the protein content gradually decreased. When 0.01–0.04 g/g TSS ZnO NPs were added, the PN content in SEPS, LB-EPS, and TB-EPS increased with the increasing ZnO NP dosage. The DNA content in the EPS was 0.27 and 0.46 mg/g TSS following the addition of 0.03 g/g TSS TiO_2_ NPs and 0.04 g/g TSS ZnO NPs, which were 19% and 72% higher than that observed in the blank sample, respectively.

### 3.2. 3D-EEM Analysis

3D-EEM fluorescent spectrometry has been successfully used to analyze soluble fluorescent substances with humic-acid-like materials, fulvic-acid-like materials, aromatic-protein-like substances, and other kinds of fluorescent materials being the main soluble fluorescent substances in water and soil [36]. This approach had also been used to analyze protein-like, humic-acid-like, and fulvic-acid-like substances in sludge EPS [37]. Most of these organic molecular structures contain a conjugated double bond, carboxyl or carbonyl group, etc. The principle of fluorescence spectroscopy is that the fluorescence intensity of a substance corresponds to different excitation and emission wavelengths and that the fluorescence intensity is proportional to the concentration of fluorescent substances [38].

**Figure 4:**
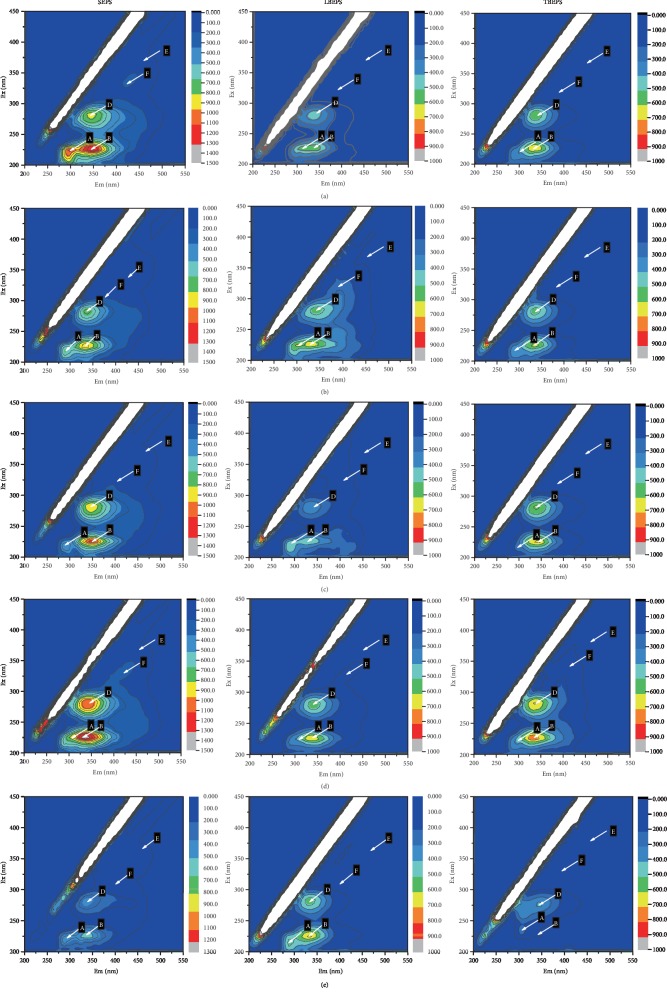
The 3D-EEMs of SEPS, LB-EPS, and TB-EPS before and after treatment with NPs: blank samples (a), Al_2_O_3_ NP-treated sludge (0.04 g/g TSS) (b), Fe_3_O_4_ NP-treated sludge (0.05 g/g TSS) (c), ZnO NP-treated sludge (0.05 g/g TSS) (d), TiO_2_ NP-treated sludge (0.03 g/g TSS) (e).

The 3D-EEM spectra of EPS before and after treatment with four kinds of NPs are shown in Figure 4. The dosage of NPs that produced the highest impact on the protein content in the EPS was selected. As presented in Figure 4(a), two more obvious fluorescence peaks B and D, along with three weak fluorescence peaks A, E, and F, appeared. Peak A at an excitation/emission wavelength (Ex/Em) of 225/300 nm corresponded to tyrosine-like proteins; peak D at Ex/Em of 280/350 nm belonged to tryptophan-like proteins [39]; peaks E and F at Ex/Em of 360/450 and 310/375 nm, respectively, corresponded to humic acid materials [15]; and Peak B at Ex/Em of 230/350 nm indicated aromatic-protein-like substances [40]. It should be emphasized that the main components of TB-EPS were almost the same as those of SEPS and LB-EPS, and the contents of tryptophan-like proteins and aromatic-protein-like substances were the highest. The main parameters of the 3D-EEM fluorescence spectra for all the samples are shown in Table 2.

As shown in Table 2, with regard to the sludge treated with Al_2_O_3_ NPs, the value of peak B and peak D decreased significantly in SEPS, that is, the concentration of tryptophan-like proteins and aromatic-protein-like substances in the SEPS was significantly reduced, which was consistent with the quantitative results (see Section 3.1). Furthermore, no cell contents were released following Al_2_O_3_ NPs treatment, suggesting that these NPs did not damage the cell wall. The content of humic-acid-like materials in each layer of EPS was similar to that in blank samples.

With regard to the sludge treated with ZnO NPs, the value of peak B and peak D increased significantly in TB-EPS and LB-EPS, that is, the concentration of tryptophan-like proteins and aromatic-protein-like substances in the TB-EPS layer and LB-EPS layer significantly increased, which was consistent with the quantitative results (see Section 3.1), indicating that the bactericidal effect of ZnO NPs destroyed the sludge cells causing the release of internal dissolved substances. In addition, the rough surface of ZnO NPs could have also caused mechanical damage to the sludge cells. Furthermore, the value of peak F increased in SEPS, that is, humic-acid-like substances in the SEPS layer significantly increased, revealing that humic-acid-like substances were mainly concentrated in the SEPS and LB-EPS [41].

With respect to the sludge treated with TiO_2_ NPs, the value of peak B, peak D, peak E, and peak F decreased, that is, the concentration of soluble organic matter in SEPS decreased, which might be owing to the photocatalytic effect of TiO_2_ NPs on the decomposition of organic matter in the SEPS. When compared with the blank samples, the value of peak B and peak D in TB-EPS decreased, that is, the concentration of tryptophan-like proteins and aromatic-protein-like substances in TB-EPS decreased, suggesting that soluble organic matter dissolved from the cells and diffused into the LB-EPS and SEPS layers, whereas the outermost layer was decomposed by the photocatalytic activity of TiO_2_ NPs.

With regard to the sludge treated with Fe_3_O_4_ NPs, the value of peak B increased in each layer, that is, the concentration of aromatic-protein-like substances in each layer of EPS increased, whereas that of tyrosine-like proteins at peak A decreased. It is worth noting that peak A of SEPS significantly weakened after treatments with the four kinds of NPs, which might be owing to the substantial impact of NPs on the properties of tyrosine-like proteins that were mainly found in LB-EPS and SEPS.

### 3.3. FT-IR Spectroscopic Analysis

The change in the main functional groups or components in EPS was detected by FT-IR spectroscopic analysis. The main components in EPS included hydrocarbons, proteins, polysaccharides, and nucleic acids. The FT-IR spectra revealed that the primary functional groups in the EPS existed in the following positions: fingerprint region (1000–600 cm^−1^), nucleic acids and carbohydrates (1200–1000 cm^−1^), amide III (1300–1220 cm^−1^), carboxylic group and hydrocarbon-like compounds (1500–1300 cm^−1^), amide II (about 1550 cm^−1^), and amide I (1700–1600 cm^−1^). Therefore, the main functional groups or components in EPS were located in the 2000–600 cm^−1^ region. As the constituents in the fingerprint region were complex and the main functional groups gathered in the range of 1000–1800 cm^−1^, further analysis of this region was performed.

**Figure 5:**
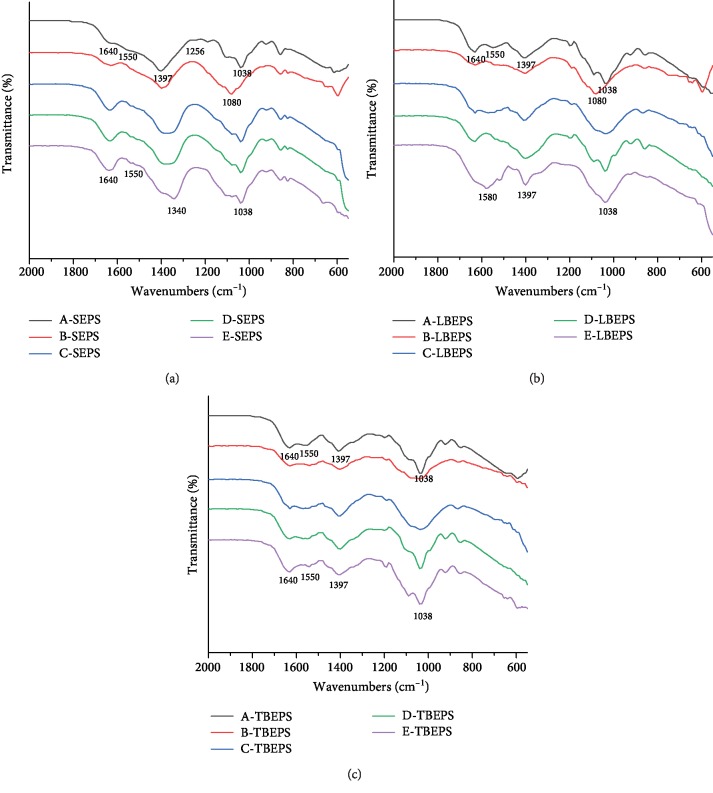
FT-IR spectra of EPS components and functional groups after treatment of concentrated sludge with different NPs: blank samples (A), Al_2_O_3_ NP-treated sludge (0.04 g/g TSS) (B), Fe_3_O_4_ NP-treated sludge (0.05 g/g TSS) (C), ZnO NP-treated sludge (0.05 g/g TSS) (D), TiO_2_ NP-treated sludge (0.03 g/g TSS) (E).

The FT-IR spectrum of SEPS extracted from the blank sample is shown in Figure 5(a). The carbohydrates were located in a strong absorption zone of 1038 cm^−1^, which was caused by the stretching vibration of C-O and C-O-C [42]. Amide III was found at 1256 cm^−1^, which was induced by the tensile vibration of C=O [17]. In addition, the absorption band at 1397 cm^−1^ was mainly caused by the bending vibration of O-H in the carboxyl group [43]. The absorption band located in the 1510 cm^−1^ region may be caused by the ring vibration of phenol, indicating the presence of tyrosine [44]. The amide I absorption band at 1640 cm^−1^ was mainly attributed to the stretching of C=O in the amide groups [17].

The FT-IR spectrum of the SEPS extracted from NP-treated samples is shown in Figure 5(a). After Al_2_O_3_ NP treatment, the intensity of the absorption peak of amide I obviously decreased at 1640 cm^−1^. On the contrary, the absorption peak of amide I at 1640 cm^−1^ increased after ZnO NP, TiO_2_ NP, and Fe_3_O_4_ NP treatments, which may be caused by higher intracellular protein dissolution. It is worth noting that after treatments with four kinds of NPs, the amide II band located at 1550 cm^−1^ decreased, which may be owing to the influence of NPs on the structure of proteins. With regard to the TiO_2_ NP-treated samples, the absorption peak located at 1397 cm^−1^ shifted to the right, which may be affected by photocatalysis, leading to some substitution reactions of O-H in the carboxyl group. After Al_2_O_3_ NP treatment, the absorption peaks of carbohydrates at 1038 cm^−1^ shifted to the left, which may be owing to the change in the type and structure of polysaccharides.

The FT-IR spectrum of LB-EPS is shown in Figure 5(b). With regard to the EPS samples treated with Al_2_O_3_ NPs, both the absorption intensity of amide I and the absorption intensity of amide II decreased, and the absorption peaks of carbohydrates located at 1038 cm^−1^ moved to the left, indicating that the species and structures of polysaccharides had changed. In other words, the changes in the functional groups in TB-EPS were similar to those in SEPS. With respect to the Fe_3_O_4_ NP-treated samples, the absorption intensity of amide II located at 1550 cm^−1^ enhanced, whereas the other functional groups did not show significant change, demonstrating that the structure of proteins was altered after Fe_3_O_4_ NP treatment. Following TiO_2_ NP treatment, the absorption intensity of both amide I and amide II increased, and the absorption peak of amide I significantly moved to the right, revealing that the structure and type of proteins changed with the increasing amount of dissolved proteins.

The FT-IR spectrum of TB-EPS is shown in Figure 5(c). With regard to samples treated with Al_2_O_3_ NPs, the absorption intensity of each peak was significantly decreased. However, the position of carbohydrate absorption peaks did not change, indicating that the effect of Al_2_O_3_ NPs on carbohydrates was only intense on SEPS and LB-EPS. With respect to the TiO_2_ NP-treated samples and ZnO NP-treated samples, the intensity of each absorption peak increased, possibly owing to the dissolution of intracellular substances.

**Figure 6:**
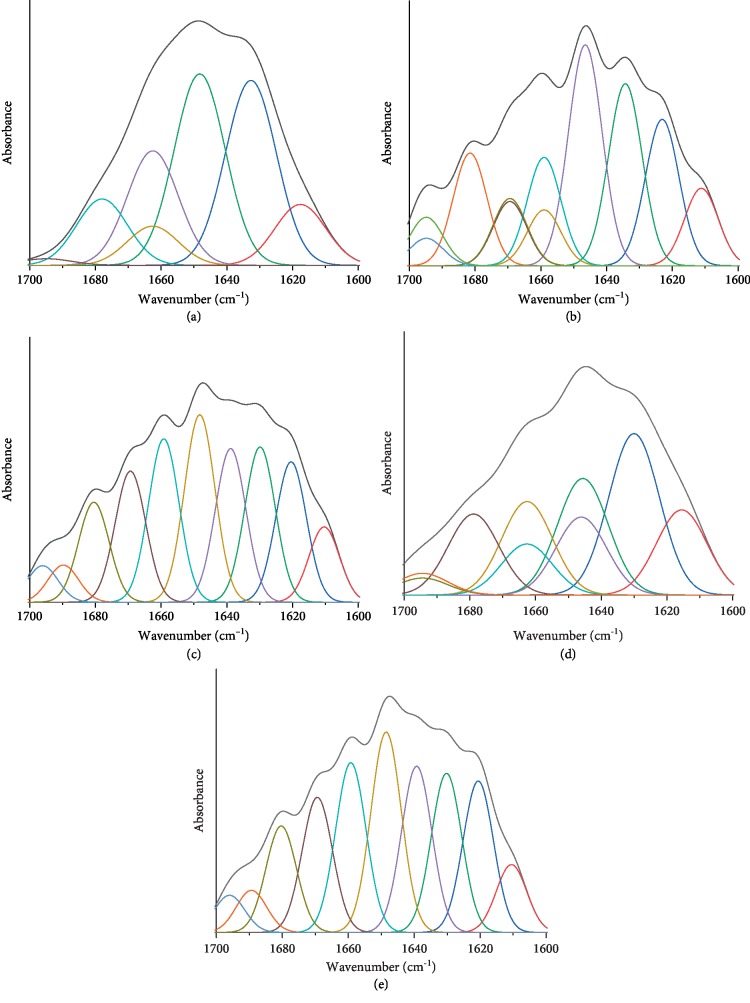
Curve fitting of second derivative spectra for EPS. Blank samples (a), Al_2_O_3_ NP-treated sludge (0.04 g/g TSS) (b), Fe_3_O_4_ NP-treated sludge (0.05 g/g TSS) (c), ZnO NP-treated sludge (0.05 g/g TSS) (d), and TiO_2_ NP-treated sludge (0.03 g/g TSS) (e).

To further explore the action of NPs on proteins in EPS, the second derivative spectrum was applied for the analysis of the ratio of each secondary structure of the proteins [20, 16]. The properties of EPS proteins were examined by spectral analysis of the reported model proteins and known structural peptides [45, 19]. The image obtained after fitting the original spectral curve is shown in Figure 6 and Figs [Supplementary-material supplementary-material-1]. The peaks located at 1700–1600 cm^−1^ were separated. The assignment of secondary structures and the differences in the composition of the secondary structures in different samples are presented in Table 3. After Al_2_O_3_ NP treatment, the percentages of *α*-helix slightly increased in the LB-EPS and TB-EPS, while the percentages of *β*-sheet also increased. However, the present study could not confirm whether the effect of high levels of *α*-helix in the EPS could lead to cell aggregation. Hou et al. [46] found that low levels of *α*-helix and high levels of *β*-sheet or random coil may lead to loose sludge structures that are not conducive to sludge dewaterability. In the present study, the value of *α*-helix/(*β*-sheet+random coil) increased from 0.513 to 0.637 in SEPS. As the protein content in SEPS accounted for 70%–80% of the total proteins in EPS, the changes in the secondary structure of proteins in SEPS can approximately represent the overall change trend of EPS. Hence, the changes in the secondary structure of proteins in SEPS were categorically analyzed.

With regard to the Fe_3_O_4_ NP-treated samples, the percentage of *α*-helix significantly increased, while that of the *β*-sheet slightly decreased in SEPS and LB-EPS. In addition, the percentage of random coli also decreased. It has been reported that aggregation, adsorption, and flocculation of microbial aggregates could be enhanced by *α*-helix and reduced by *β*-sheet [47, 19], suggesting that the aggregation, adsorption, and flocculation of microbial aggregates could be enhanced by Fe_3_O_4_ NP treatment.

After TiO_2_ NP treatment, the value of *α*-helix/(*β*-sheet+random coil) decreased from 0.513 to 0.383 in SEPS, indicating that TiO_2_ NP treatment is not conducive to the aggregation, adsorption, and flocculation of microbial aggregates. With regard to the ZnO NP-treated samples, both the percentages of *β*-sheet and *α*-helix decreased, but the value of *α*-helix/(*β*-sheet+random coil) slightly increased from 0.513 to 0.557 in SEPS. It must be noted that the percentages of 3-turn helix increased in SEPS, LB-EPS, and TB-EPS. Previous studies have reported that aggregation, adsorption, and biofilm formation of bacterial cells could be enhanced by certain protein secondary structures, such as aggregated strands, *β*-sheet, and *α*-helix and 3-turn helices; however, these factors could be suppressed by antiparallel *β*-structures and random coils [16, 17]. Therefore, although ZnO NPs can significantly increase the protein content in EPS, it has a little effect on the secondary structure of proteins in EPS. While the present study focused on the impact of NPs on the quantity and quality of EPS proteins, in future research, TEM images will be used to characterize the cell state after the interaction between sludge and NPs.

## 4. Conclusion

1. When the concentrated sludge was treated with TiO_2_ NPs, Fe_3_O_4_ NPs, and ZnO NPs, the protein content first increased and then decreased with the increase of the dosage of NPs. While when the concentrated sludge was treated with Al_2_O_3_ NPs, the protein content first decreased and then remained stable with the increase of the dosage of NPs. In addition, the protein distribution in the EPS of the sludge treated with NPs will also change to some extent
2. When the concentrated sludge was treated with Al_2_O_3_ NPs, the concentration of tryptophan-like proteins and aromatic-protein-like substances decreased. The concentration of tyrosine-like protein in the EPS of concentrated sludge decreased after the treatment of four kinds of nanoparticles
3. Analysis of the secondary structure of proteins revealed that the aggregation, adsorption, and flocculation of microbial aggregates could be enhanced by Fe_3_O_4_ NP treatment and weakened by TiO_2_ NP treatment

## Figures and Tables

**Table 1:** Characteristics of the different types of sludge.

Sludge types	Water contents (%)	pH	TSS^∗^ (%)	Ash (%)	VSS^∗∗^ (%)
Concentrated sludge	97	6.8	3	1.2	1.8
Mechanical dewatered sludge	85	7.0	15	4.0	11.0

^∗^TSS = total suspended solid, ^∗∗^VSS = volatile suspended solid.

**Table 2:** Main parameters of the 3D-EEM fluorescence spectra for all the samples.

	SEPS				LB-EPS				TB-EPS			
	Peak B	Peak D	Peak E	Peak F	Peak B	Peak D	Peak E	Peak F	Peak B	Peak D	Peak E	Peak F
*λ*_ex/em_	225/350	280/350	360/450	310/375	225/350	280/350	360/450	310/375	225/350	280/350	360/450	310/375
a	1297	822	188	160	484	452	52	51	386	579	97	41
b	931	747	191	154	643	540	56	62	630	544	94	44
c	1354	701	197	211	504	319	46	136	721	567	80	141
d	823	1387	200	278	716	317	60	97	774	336	88	72
e	797	366	186	150	105	422	67	79	576	716	63	72

**Table 3:** Effects of NPs on EPS protein secondary structures in different EPS fractions.

EPS fractions	NPs	Secondary structures (%)					
		Aggregated strands	*β*-Sheet	Random coli	*α*-Helix	3-turn helix	Antiparallel *β*-sheet/aggregated strands
		1610-1625 cm^−1^	1630-1640 cm^−1^	1640-1645 cm^−1^	1648-1657 cm^−1^	1659-1972 cm^−1^	1680-1695 cm^−1^
SEPS	Blank	14.58%	17.70%	16.86%	17.74%	15.75%	17.37%
	Al_2_O_3_	20.25%	17.85%	9.66%	17.55%	18.59%	16.10%
	Fe_3_O_4_	19.32%	17.19%	12.06%	21.08%	18.18%	12.17%
	ZnO	17.83%	13.34%	13.84%	15.14%	24.48%	15.37%
	TiO_2_	16.67%	21.04%	11.48%	12.46%	25.77%	12.59%

LB-EPS	Blank	13.31%	22.52%	21.75%	19.49%	12.50%	10.42%
	Al_2_O_3_	16.71%	22.56%	21.72%	25.06%	25.63%	12.08%
	Fe_3_O_4_	19.30%	18.13%	9.74%	24.58%	12.67%	13.08%
	ZnO	16.18%	14.63%	13.64%	15.42%	25.14%	11.89%
	TiO_2_	20.02%	23.26%	9.59%	15.96%	16.01%	12.09%

TB-EPS	Blank	15.54%	15.87%	15.65%	17.1%	26.00%	9.79%
	Al_2_O_3_	15.26%	20.36%	11.01%	19.15%	18.41%	12.13%
	Fe_3_O_4_	11.40%	23.67%	11.40%	26.68%	14.30%	10.53%
	ZnO	14.85%	13.11%	14.25%	16.07%	27.91%	13.80%
	TiO_2_	10.26%	14.27%	13.84%	15.24%	13.05%	5.71%

## Data Availability

The [DATA TYPE] data used to support the findings of this study are included within the article and supplementary information files.

## Supplementary Materials

Supplementary Materials S1: characterization of NPs: SEM image and EDS spectrum of nanoparticles. S2: characterization of NPs: XRD patterns and average size of nanoparticles. S3: curve fitting of second derivative spectra for TB-EPS and LB-EPS.

## References

[1] Kiser M. A., Westerhoff P., Benn T., Wang Y., Pérez-Rivera J., Hristovski K. (2009). Titanium nanomaterial removal and release from wastewater treatment plants. *Environmental Science & Technology*.

[2] Sheng G. P., Yu H. Q., Li X. Y. (2010). Extracellular polymeric substances (eps) of microbial aggregates in biological wastewater treatment systems: a review. *Biotechnology Advances*.

[3] Poxon T. L., Darby J. L. (1997). Extracellular polyanions in digested sludge: measurement and relationship to sludge dewaterability. *Water Research*.

[4] Mikkelsen L. H., Keiding K. (2002). Physico-chemical characteristics of full scale sewage sludges with implications to dewatering. *Water Research*.

[5] Frølund B., Palmgren R., Keiding K., Nielsen P. H. (1996). Extraction of extracellular polymers from activated sludge using a cation exchange resin. *Water Research*.

[6] Herbst F. A., Lünsmann V., Kjeldal H. (2016). Enhancing metaproteomics-the value of models and defined environmental microbial systems. *Proteomics*.

[7] Higgins M. J., Novak J. T. (1997). The effect of cations on the settling and dewatering of activated sludges: laboratory results. *Water Environment Research*.

[8] Zhao J., Xin M., Zhang J. (2020). Diclofenac inhibited the biological phosphorus removal: performance and mechanism. *Chemosphere*.

[9] Hou J., Miao L., Wang C., Wang P., Ao Y., Lv B. (2015). Effect of CuO nanoparticles on the production and composition of extracellular polymeric substances and physicochemical stability of activated sludge flocs. *Bioresource Technology*.

[10] He Q., Yuan Z., Zhang J. (2017). Insight into the impact of ZnO nanoparticles on aerobic granular sludge under shock loading. *Chemosphere*.

[11] Cervantes-Avilés P., Díaz Barriga-Castro E., Palma-Tirado L., Cuevas-Rodríguez G. (2017). Interactions and effects of metal oxide nanoparticles on microorganisms involved in biological wastewater treatment. *Microscopy Research and Technique*.

[12] Cervantes-Avilés P., Ida J., Toda T., Cuevas-Rodríguez G. (2018). Effects and fate of TiO_2_ nanoparticles in the anaerobic treatment of wastewater and waste sludge. *Journal of Environmental Management*.

[13] Wiesner M. R., Lowry G. V., Jones K. L. (2009). Decreasing uncertainties in assessing environmental exposure, risk, and ecological implications of nanomaterials. *Environmental Science & Technology*.

[14] Peymani-Motlagh S. M., Moeinian N., Rostami M. (2019). Effect of Gd^3+^-, Pr^3+^- or Sm^3+^-substituted cobalt-zinc ferrite on photodegradation of methyl orange and cytotoxicity tests. *Journal of Rare Earths*.

[15] Wei D., Wang Y., Wang X. (2015). Toxicity assessment of 4-chlorophenol to aerobic granular sludge and its interaction with extracellular polymeric substances. *Journal of Hazardous Materials*.

[16] Beech I., Hanjagsit L., Kalaji M., Neal A. L., Zinkevich V. (1999). Chemical and structural characterization of exopolymers produced by Pseudomonas sp. NCIMB 2021 in continuous culture. *Microbiology*.

[17] Badireddy A. R., Chellam S., Gassman P. L., Engelhard M. H., Lea A. S., Rosso K. M. (2010). Role of extracellular polymeric substances in bioflocculation of activated sludge microorganisms under glucose-controlled conditions. *Water Research*.

[18] Wang W., Liu W., Wang L., Yang T., Li R. (2016). Characteristics and distribution research on extracellular polymer substance extracted from sewage sludge. *Journal of Environmental Biology*.

[19] Yin C., Meng F., Chen G. H. (2015). Spectroscopic characterization of extracellular polymeric substances from a mixed culture dominated by ammonia-oxidizing bacteria. *Water Research*.

[20] Buijs J., Norde W., Lichtenbelt J. W. T. (1996). Changes in the secondary structure of adsorbed IgG and F(ab‘)2Studied by FTIR spectroscopy. *Langmuir*.

[21] Nielsen P. H., Jahn A. (1999). Extraction of EPS. *Microbial Extracellular Polymeric Substances*.

[22] Yu G. H., He P. J., Shao L. M. (2009). Characteristics of extracellular polymeric substances (EPS) fractions from excess sludges and their effects on bioflocculability. *Bioresource Technology*.

[23] Jones B., Renaut R. W. (2007). Selective mineralization of microbes in Fe-rich precipitates (jarosite, hydrous ferric oxides) from acid hot springs in the Waiotapu geothermal area, North Island, New Zealand. *Sedimentary Geology*.

[24] Brayner R., Ferrari-Iliou R., Brivois N., Djediat S., Benedetti M. F., Fiévet F. (2006). Toxicological impact studies based on Escherichia coli bacteria in ultrafine ZnO nanoparticles colloidal medium. *Nano Letters*.

[25] Padmavathy N., Vijayaraghavan R. (2008). Enhanced bioactivity of ZnO nanoparticles—an antimicrobial study. *Science and Technology of Advanced Materials*.

[26] Xu S., Wang Z. L. (2011). One-dimensional ZnO nanostructures: solution growth and functional properties. *Nano Research*.

[27] Maness P.-C., Smolinski S., Blake D. M., Huang Z., Wolfrum E. J., Jacoby W. A. (1999). Bactericidal activity of photocatalytic TiO_2_ reaction : toward an understanding of its killing mechanism. *Applied and Environmental Microbiology*.

[28] Jiang J., Oberdorster G., Biswas P. (2009). Characterization of size, surface charge, and agglomeration state of nanoparticle dispersions for toxicological studies. *Journal of Nanoparticle Research*.

[29] Rottman J., Shadman F., Sierra-Alvarez R. (2012). Interactions of inorganic oxide nanoparticles with sewage biosolids. *Water Science & Technology*.

[30] Kim I. S., Baek M., Choi S. J. (2010). Comparative cytotoxicity of Al_2_O_3_, CeO_2_, TiO_2_ and ZnO nanoparticles to human lung cells. *Journal of Nanoscience and Nanotechnology*.

[31] Kocbek P., Teskač K., Kreft M. E., Kristl J. (2010). Toxicological aspects of long-term treatment of keratinocytes with ZnO and TiO_2_ nanoparticles. *Small*.

[32] Sadiq I. M., Chowdhury B., Chandrasekaran N., Mukherjee A. (2009). Antimicrobial sensitivity of *Escherichia coli* to alumina nanoparticles. *Nanomedicine: Nanotechnology, Biology and Medicine*.

[33] Yang S., Sheng G., Montavon G. (2013). Investigation of Eu(III) immobilization on *γ*-Al_2_O_3_ surfaces by combining batch technique and exafs analyses: role of contact time and humic acid. *Geochimica et Cosmochimica Acta*.

[34] Wang H., Wick R. L., Xing B. (2009). Toxicity of nanoparticulate and bulk ZnO, Al_2_O_3_ and TiO_2_ to the nematode caenorhabditis elegans. *Environmental Pollution*.

[35] Absalan G., Asadi M., Kamran S., Sheikhian L., Goltz D. M. (2011). Removal of reactive red-120 and 4-(2-pyridylazo) resorcinol from aqueous samples by Fe_3_O_4_ magnetic nanoparticles using ionic liquid as modifier. *Journal of Hazardous Materials*.

[36] Coble P. G. (1996). Characterization of marine and terrestrial DOM in seawater using excitation-emission matrix spectroscopy. *Marine Chemistry*.

[37] Sheng G. P., Yu H.-Q. (2006). Characterization of extracellular polymeric substances of aerobic and anaerobic sludge using three-dimensional excitation and emission matrix fluorescence spectroscopy. *Water Research*.

[38] Henderson R. K., Baker A., Murphy K. R., Hambly A., Stuetz R. M., Khan S. J. (2009). Fluorescence as a potential monitoring tool for recycled water systems: a review. *Water Research*.

[39] Chen W., Westerhoff P., Leenheer J. A., Booksh K. (2003). Fluorescence excitation-emission matrix regional integration to quantify spectra for dissolved organic matter. *Environmental Science & Technology*.

[40] Wang Z., Gao M., Wang Z. (2013). Effect of salinity on extracellular polymeric substances of activated sludge from an anoxic–aerobic sequencing batch reactor. *Chemosphere*.

[41] Yu G.-H., He P.-J., Shao L.-M. (2010). Novel insights into sludge dewaterability by fluorescence excitation-emission matrix combined with parallel factor analysis. *Water Research*.

[42] Bramhachari P. V., Kishor P. B., Ramadevi R., Kumar R., Rao B. R., Dubey S. K. (2007). Isolation and characterization of mucous exopolysaccharide (EPS) produced by Vibrio furnissii strain VB0S3. *Journal of Microbiology and Biotechnology*.

[43] Small G. W. (1992). Spectrometric identification of organic compounds. *Vibrational Spectroscopy*.

[44] Barth A., Zscherp C. (2002). What vibrations tell us about proteins. *Quarterly Reviews of Biophysics*.

[45] Badireddy A. R., Korpol B. R., Chellam S. (2008). Spectroscopic characterization of extracellular polymeric substances from Escherichia coli and Serratia marcescens: suppression using sub-inhibitory concentrations of bismuth thiols. *Biomacromolecules*.

[46] Hou X., Liu S., Zhang Z. (2015). Role of extracellular polymeric substance in determining the high aggregation ability of anammox sludge. *Water Research*.

[47] Omoike A., Chorover J. (2004). Spectroscopic study of extracellular polymeric substances from Bacillus subtilis: aqueous chemistry and adsorption effects. *Biomacromolecules*.

