# Supplementary material for: Effects of Four Kinds of Oxide Nanoparticles on Proteins in Extracellular Polymeric Substances of Sludge
Source: Biomed Res Int. 2020 Mar 2;2020:1754134. doi: 10.1155/2020/1754134 (PMC7072109; doi:10.1155/2020/1754134)
Supplement: Supplementary Materials — S1: characterization of NPs: SEM image and EDS spectrum of nanoparticles. S2: characterization of NPs: XRD patterns and average size of nanoparticles. S3: curve fitting of second derivative spectra for TB-EPS and LB-EPS. [file 1754134.f1.pdf]

Supplementary materials

Manuscript title：Effects of Four Kinds of Oxide Nanoparticles on Protein in EPS of Sludge

Complete List of Authors:

Weiyun Wang*; College of Energy and Environment, Shenyang Aerospace University, Shenyang 110136, China; Key Laboratory of Clean Energy, Liaoning Province, 110136, China.

Ying Zang; College of Energy and Environment, Shenyang Aerospace University, Shenyang 110136, China; Key Laboratory of Clean Energy, Liaoning Province, 110136, China.

Cijia Wang; College of Energy and Environment, Shenyang Aerospace University, Shenyang 110136, China; Key Laboratory of Clean Energy, Liaoning Province, 110136, China.

Kangni Wang; College of Energy and Environment, Shenyang Aerospace University, Shenyang 110136, China; Key Laboratory of Clean Energy, Liaoning Province, 110136, China.

Rundong Li; College of Energy and Environment, Shenyang Aerospace University, Shenyang 110136, China; Key Laboratory of Clean Energy, Liaoning Province, 110136, China.

Correspondent Author: Weiyun Wang;

E-mail: cathywwy@sau.edu.cn

Address: No.37 Daoyi South Avenue, Shenbei New Area,Shenyang, China 110136

Part S1 Characterization of NPs: SEM image and EDS spectrum of nanoparticles

(a) SEM image of Al_2O_3 nanoparticles (NPs)

(b) EDS spectrum of Al_2O_3 nanoparticles (NPs)

(c) SEM image of Fe_3O_4 NPs

(d) EDS spectrum of Fe_3O_4 NPs

(e) SEM image of ZnO NPs

(f) EDS spectrum of ZnO NPs

(g) SEM image of TiO_2 NPs

(h) EDS spectrum of TiO_2 NPs

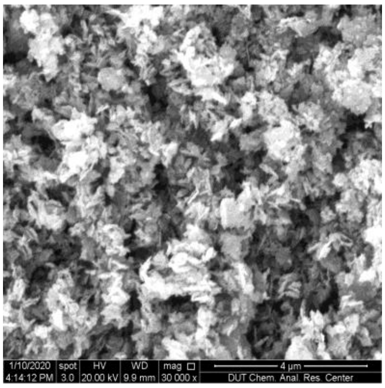

(a)

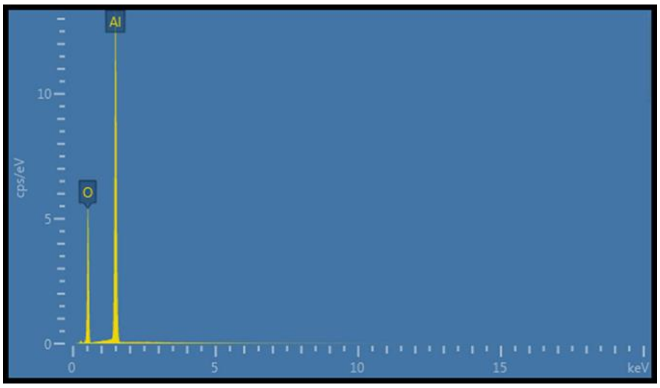

(b)

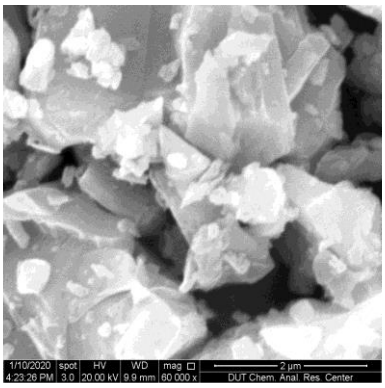

(c)

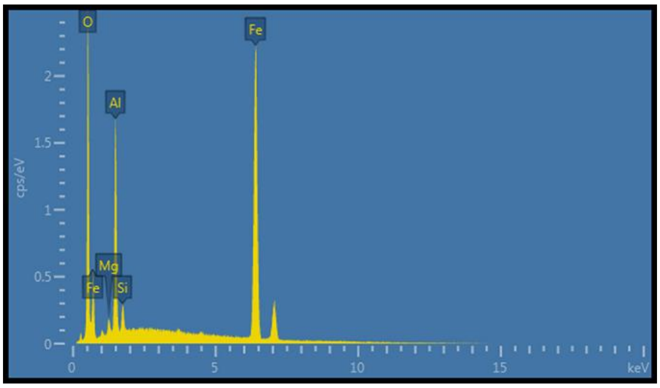

(d)

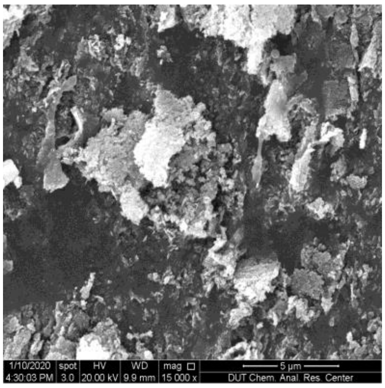

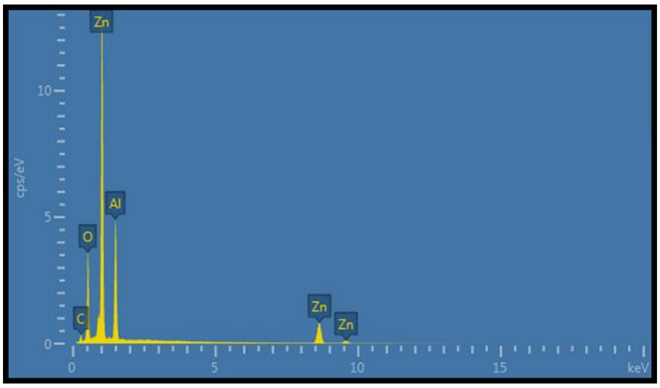

(e) (f)

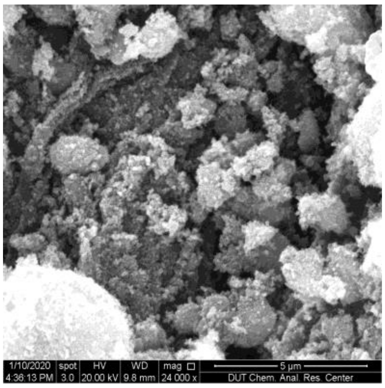

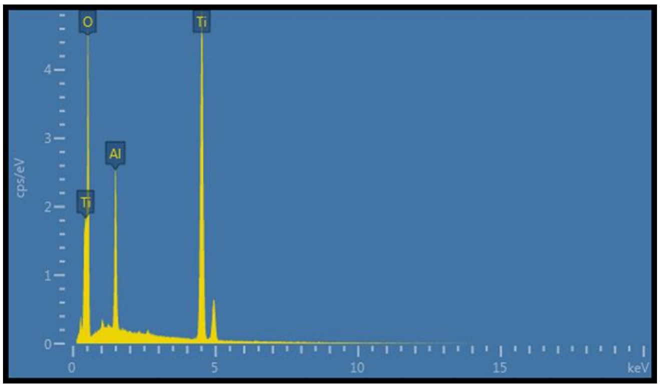

(g) (h)

Fig. S1. The SEM image (a) and EDS spectrum (b) of Al_2O_3 nanoparticles (NPs), The SEM image (c) and EDS spectrum (d) of Fe_3O_4 NPs , The SEM image (e) and EDS spectrum (f) of ZnO NPs, The SEM image (g) and EDS spectrum (h) of TiO_2 NPs.

Part S2 Characterization of NPs: XRD patterns and average size of nanoparticles

XRD patterns was analyzed by jade 9.0 software, after calculation, the particle size of Fe_3O_4 NPs is larger given 30nm, and the sizes of other nanoparticles are almost 30nm.

(a) XRD pattern of Al_2O_3 nanoparticles (NPs), the average size of Al_2O_3 NPs is 34nm.

(b) XRD pattern of Fe_3O_4 NPs, the average size of Fe_3O_4 NPs is 92 nm.

(c) XRD pattern of ZnO NPs, the average size of ZnO NPs is 25 nm.

(d) XRD pattern of TiO_2 NPs, the average size of TiO_2 NPs is 23 nm.

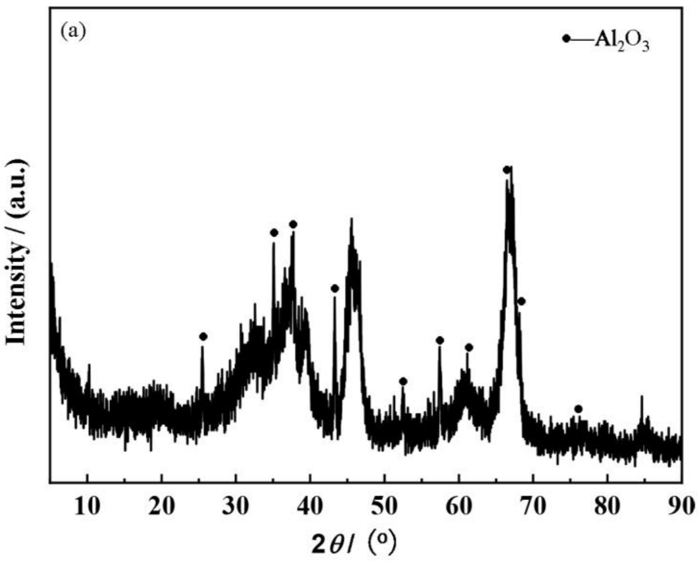

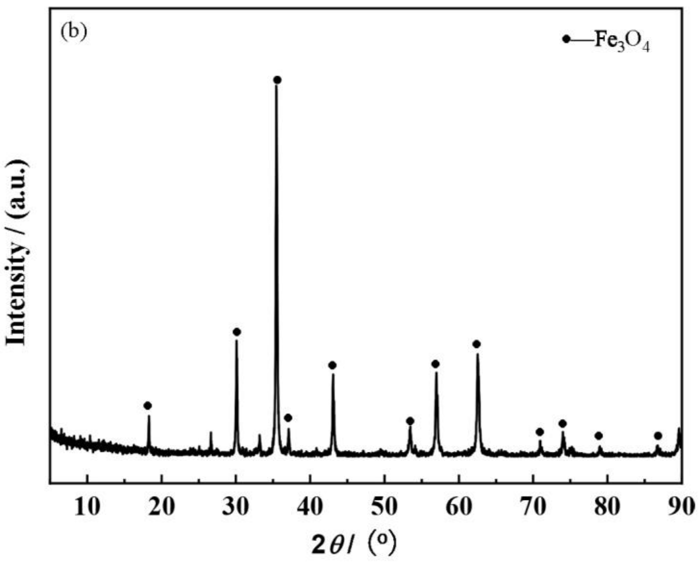

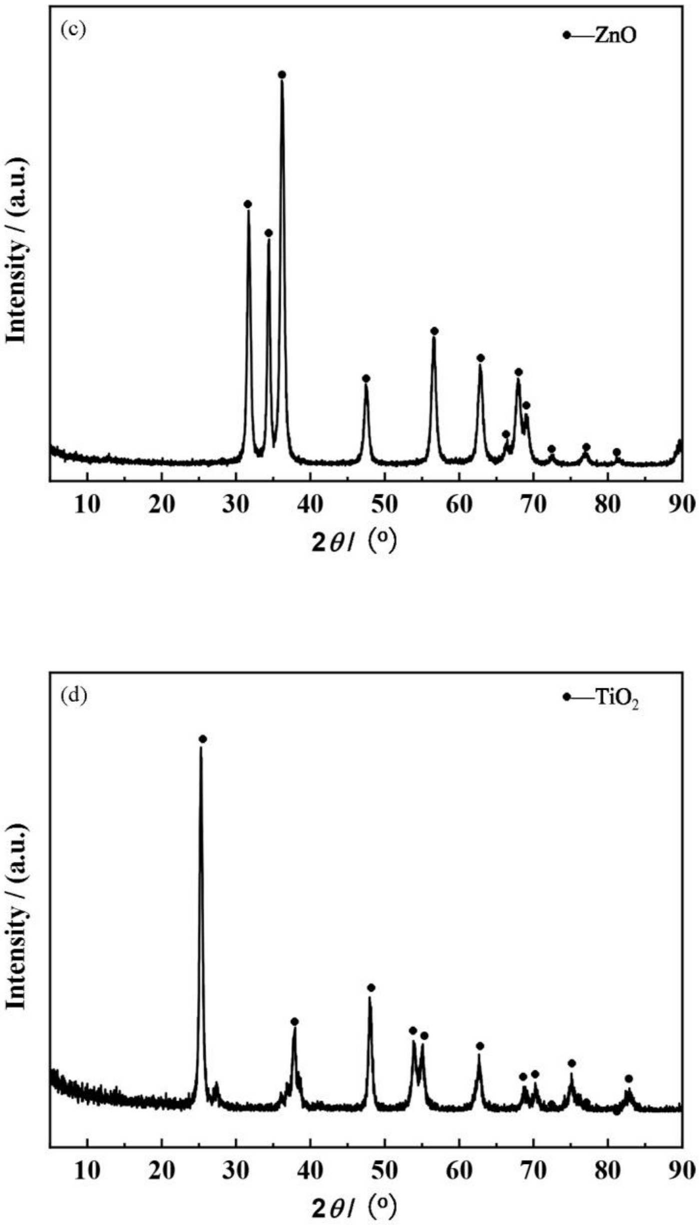

Fig. S2. XRD pattern of Al_2O_3 nanoparticles (NPs) (a), XRD pattern of Fe_3O_4 NPs (b), XRD pattern of ZnO NPs (c), XRD pattern of TiO_2 NPs (d).

Part S3 Curve-fitting of second derivative spectra for TB-EPS and LB-EPS

0-LB Curve-fitting of second derivative spectra for LB-EPS.

0-TB Curve-fitting of second derivative spectra for TB-EPS.

a-LB When sludge is treated with 0.04g/g TSS Al_2O_3 NPs, the Curve-fitting of second derivative spectra for LB-EPS.

a-TB When sludge is treated with 0.05g/g TSS Al_2O_3 NPs, the Curve-fitting of second derivative spectra for TB-EPS.

b-LB When sludge is treated with 0.05g/g TSS Fe_3O_4 NPs, the Curve-fitting of second derivative spectra for LB-EPS.

b-TB When sludge is treated with 0.05g/g TSS Fe_3O_4 NPs, the Curve-fitting of second derivative spectra for TB-EPS.

c-LB When sludge is treated with 0.05g/g TSS ZnO NPs, the Curve-fitting of second derivative spectra for LB-EPS.

c-TB When sludge is treated with 0.05g/g TSS ZnO NPs, the Curve-fitting of second derivative spectra for TB-EPS.

d-LB When sludge is treated with 0.03g/g TSS TiO_2 NPs, the Curve-fitting of second derivative spectra for LB-EPS.

d-TB When sludge is treated with 0.03g/g TSS TiO_2 NPs, the Curve-fitting of second derivative spectra for TB-EPS.

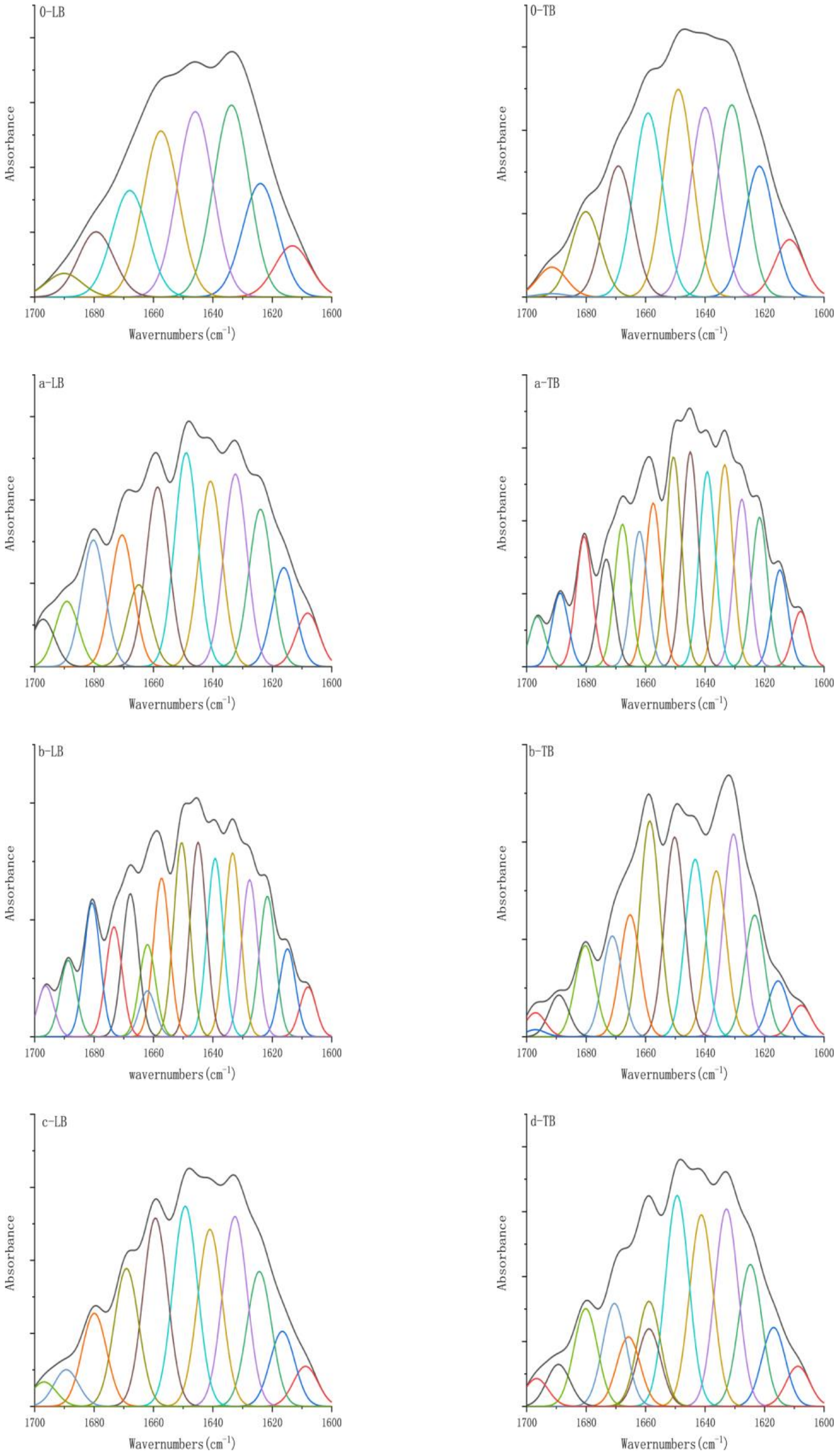
0-LB
0-TB
a-LB
a-TB
b-LB
b-TB
c-LB
d-TB
Absorbance
Wavernumbers (cm^{-1})
1700
1680
1660
1640
1620
1600

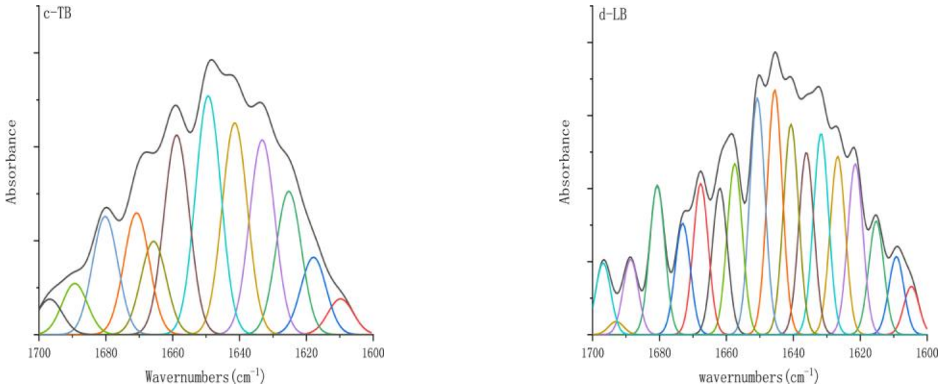

Fig. S3. Curve-fitting of second derivative spectra for extracellular polymeric substances (EPS). Blank samples (0), Al_2O_3 nanoparticles (NPs) treated sludge (0.04 g/g TSS) (a), Fe_3O_4 NPs treated sludge (0.05 g/g TSS) (b), ZnO NPs treated sludge (0.05 g/g TSS) (c), TiO_2 NPs treated sludge (0.03 g/g TSS) (d). TSS = total suspended solid.
